# Distinct Infiltration of T Cell Populations in Bladder Cancer Molecular Subtypes

**DOI:** 10.3390/cells13110926

**Published:** 2024-05-28

**Authors:** Viktor Sincic, Ken F. Arlenhold, Sarah Richtmann, Henrik Lilljebjörn, Pontus Eriksson, Gottfrid Sjödahl, Mats Wokander, Karin Hägerbrand, Peter Ellmark, Thoas Fioretos, Carl A. K. Borrebaeck, Fredrik Liedberg, Kristina Lundberg

**Affiliations:** 1 Department of Immunotechnology, Lund University, 223 81 Lund, Sweden; 2 CREATE Health Cancer Center, Lund University, 223 81 Lund, Sweden; 3 Division of Clinical Genetics, Department of Laboratory Medicine, Lund University, 221 84 Lund, Sweden; 4 Division of Oncology, Department of Clinical Sciences Lund, Lund University, 221 84 Lund, Sweden; 5 Department of Translational Medicine, Lund University, 205 02 Malmö, Sweden; 6 Department of Urology, Skåne University Hospital, 205 02 Malmö, Sweden; 7 Alligator Bioscience AB, Medicon Village, 223 63 Lund, Sweden; 8 Department of Clinical Genetics, University and Regional Laboratories Region Skåne, 221 85 Lund, Sweden

**Keywords:** bladder cancer, molecular subtypes, T cell population, exhaustion, immunotherapy

## Abstract

Bladder cancer is a heterogenous disease, and molecular subtyping is a promising method to capture this variability. Currently, the immune compartment in relation to subtypes is poorly characterized. Here, we analyzed the immune compartment in bladder tumors and normal bladder urothelium with a focus on T cell subpopulations using flow cytometry and RNA sequencing. The results were investigated in relation to tumor invasiveness (NMIBC/MIBC) and molecular subtypes according to the Lund Taxonomy system. Whereas the NMIBC/MIBC differed in the overall immune infiltration only, the molecular subtypes differed both in terms of immune infiltration and immune compartment compositions. The Basal/Squamous (Ba/Sq) and genomically unstable (GU) tumors displayed increased immune infiltration compared to urothelial-like (Uro) tumors. Additionally, the GU tumors had a higher proportion of regulatory T cells within the immune compartment compared to Uro tumors. Furthermore, sequencing showed higher levels of exhaustion in CD8^+^ T cells from GU tumors compared to both Uro tumors and the control. Although no such difference was detected at the transcriptomic level in Uro tumors compared to the controls, CD8^+^ T cells in Uro tumors showed higher expression of several exhaustion markers at the protein level. Taken together, our findings indicate that depending on the molecular subtype, different immunotherapeutic interventions might be warranted.

## 1. Introduction

In 2020, 573,000 patients worldwide were diagnosed with bladder cancer, and 213,000 succumbed to the disease, thus making bladder cancer the second most prevalent urogenital malignant disease globally [1]. Bladder cancer is traditionally divided into non-muscle-invasive bladder cancer (NMIBC) and muscle-invasive bladder cancer (MIBC). Around 25% of bladder cancer patients have muscle-invasive disease at diagnosis, and despite drastic treatment, including neoadjuvant chemotherapy plus radical cystectomy, and sometimes also adjuvant immunotherapy, the 5-year survival rate is only around 50% [2,3]. Patients diagnosed with NMIBC have a better 5-year survival rate, but approximately every second patient suffers from at least one local recurrence, and, in the long term, there is a risk of progression to MIBC [4]. The high recurrence rate in NMIBC urges frequent monitoring and multiple treatment occasions, making bladder cancer one of the most expensive cancers per patient for society [5]. Treatment of bladder cancer has largely remained the same during the last decades, and survival has not improved to the same extent as in many other cancer forms [2].

One reason for the lack of progress is the extensive heterogeneity of bladder cancer and the inability of the traditional classification to capture this, leading to low-resolution patient stratification. Molecular classification systems based on gene expression profiling have been developed to account for disease heterogeneity and thereby better stratify patients. The Lund Taxonomy molecular classification system is solely based on cancer cells, and it is the only classification system that is applicable to both NMIBC and MIBC [6]. The molecular subtypes include, e.g., the urothelial-like (Uro), Basal/Squamous (Ba/Sq), and genomically unstable (GU) subtypes, and these have been shown to differentially respond to therapy [7]. For example, the response rate to immunotherapy has been shown to be around 50% for the GU subtype, whereas the Uro and Ba/Sq subtypes had an overall response rate of around 20% [8].

It is additionally well-established that the immune microenvironment has a major impact on bladder cancer. For example, CD8^+^ T cells in the tumor have been shown to correlate with overall survival in MIBC [9]. In contrast, regulatory T cells (Tregs) are generally known as tumor-promoting cells in cancers, including bladder cancer [10]. However, there are also studies showing that infiltration of Tregs correlates with improved survival of bladder cancer patients [11,12]. The infiltration of specific immune cells also impacts the response to immunotherapy, and several studies have, for example, demonstrated that the presence of CD8^+^ T cells in tumors correlate with the response [8,13]. Despite some efforts, the immune microenvironment of the different molecular subtypes is still poorly understood. So far, investigations have mainly relied on bulk RNA sequencing data and tissue staining using a limited set of markers in parallel [8,14,15]. Detailed studies where distinct immune populations are precisely defined in relation to specific cancer cell molecular subtypes are lacking.

In this study, we investigated the immune compartment in bladder tumors stratified according to invasiveness and molecular subtypes using the Lund Taxonomy system [7]. We assessed the infiltration of CD45^+^ immune cells, as well as the relative abundance of helper T cells (Th), Tregs, and cytotoxic CD8^+^ T cells. Additionally, we assessed transcriptomic differences of CD8^+^ T cells in GU and Uro tumors. We show that the molecular subtypes have different levels of immune infiltration as well as different cellular composition of the immune compartment. These findings indicate that bladder cancer molecular subtypes shape characteristic immune microenvironments. Dissecting and understanding these different environments could aid patient stratification and treatment choice, especially regarding immunotherapies.

## 2. Materials and Methods

### 2.1. Sample Preparation and Classification

Tumor tissue samples were cold-cup biopsies obtained from the exophytic part of the tumor during transurethral resection (TURB) of treatment-naïve patients with primary bladder cancer. Tumors were classified according to the Lund Taxonomy based on IHC and/or bulk RNA seq data as part of the UROSCANSEQ prospective sequencing initiative. Non-malignant control bladder tissues were obtained during open partial cystectomy for benign conditions or non-urothelial tumors (patient characteristics presented in Table 1). Immediately after surgery, biopsies were placed in tissue storage solution (Miltenyi Biotec, Somerville, MA, USA) and transported to the laboratory on ice. Subsequently, tissue samples were cut into small pieces in RPMI 1640 medium (Thermo Fisher Scientific, Bremen, Germany) supplemented with 0.1 mg/mL gentamycin (Sigma Aldrich, St Louis, MO, USA) and prepared into single-cell suspensions through enzymatic digestion at 37 °C for 20 min using Collagenase IV (Sigma Aldrich) (2 mg/mL) and DNase I (Sigma Aldrich) (200 Kunitz units/mL), followed by filtering through 70 µm cell strainer (BD Biosciences, San Jose, CA, USA). The study was approved by the Research Ethics Board of Lund University (Dnr 2017/269 and 2018/963) and Stockholm University (Dnr 2020-05559 and 2022-03081-02).

### 2.2. Flow Cytometry, Sorting, RNA Extraction, and Sequencing

**Figure 1:**
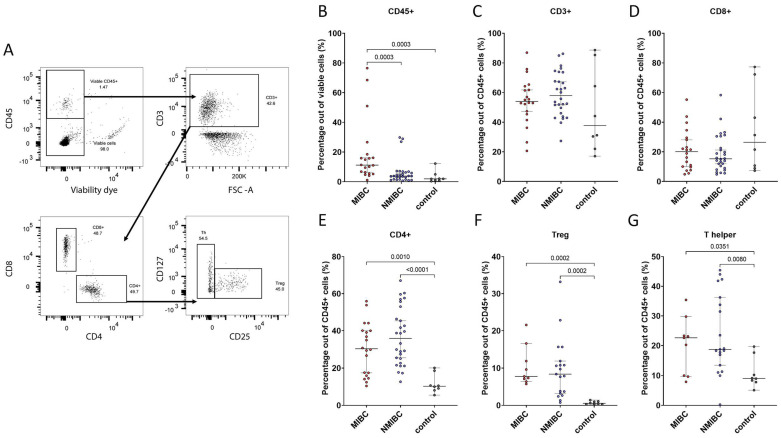
Gating strategy for analysis of Tregs, Th cells, and CD8^+^ T cells (**A**). Immune cell infiltration measured as CD45^+^ out of all viable cells (**B**). Frequency of T cells and T cell subsets out of all CD45^+^ cells (**C**–**G**). Treg = regulatory T cells, Th = T helper, NMIBC = non-muscle-invasive bladder cancer, MIBC = muscle-invasive bladder cancer. Statistical significance was determined by the Kruskal–Wallis test followed by the two-stage linear step-up procedure of Benjamini, Krieger, and Yekutieli post-test.

Cells were stained with Fixable Viability Stain (FVS) 620 (BD Biosciences) according to manufacturer’s protocol to enable viability evaluation. Cells were then washed, blocked using mouse IgG (Jackson ImmunoResearch, West Grove, PA), and finally stained with an antibody panel for 20 min at +4 °C in Brilliant Stain Buffer (BD Biosciences) (Table A1). Stained cells were acquired on a BD FACS Aria II (BD Biosciences) for analysis and sorting of CD8^+^ T cells, Th cells, and Tregs. For the phenotypic analysis, doublets were excluded, and viable T cells (FVS^−^ CD45^+^CD3^+^) were gated. CD8^+^ T cells, Tregs, and Th cells were subsequently identified as CD8^+^CD4^−^, CD4^+^CD25^+^CD127^−/dim^, and CD4^+^CD25^−^, respectively (Figure 1A). For sorting, cells expressing lineage (Lin) markers (CD14, CD16, CD19, CD20, CD56, and CD66b) were excluded before gating CD8^+^ T cells, as described above. T cell populations were sorted from tumors as well as from non-malignant control tissues from patients undergoing bladder surgery for reasons other than bladder cancer (purity > 95%). Cells were sorted directly into extraction buffer from the Arcturus PicoPure RNA Isolation Kit (Thermo Fisher Scientific). RNA sequencing was performed as previously described [16].

### 2.3. Bulk RNA Seq Analysis of Sorted CD8^+^ T Cells

RNA sequencing reads were aligned to the human reference genome hg19 using STAR 2.5.0a [17]. Reads within genes were counted using featureCounts (version 1.6.3) [18]. In brief, a gene was considered detected at read counts >5, and samples with <7000 detected genes were excluded from the analysis. Genes with >1 counts per million reads mapped (CPM) in 2 or more samples were kept for further analysis using EdgeR (version 3.38.2) [19]. Data normalization and differential gene expression analysis were carried out using DESeq2 (version 1.36.0) with the apeglm shrinkage estimator [20,21]. Differentially expressed genes, defined as adjusted *p*-values (padj) < 0.05 and absolute log2 fold change >1, were extracted. Gene set enrichment analysis (GSEA) is a method to investigate the relative enrichment of a set of genes between groups. Here, we used GSEA to compare T cell exhaustion between the molecular subtypes. The fgsea R package (version 1.22.0) with genes ranked according to the log2 fold changes obtained from the differential expression analysis was used to perform GSEA [22]. Enrichment analysis was performed with the differentially expressed genes (padj < 0.05 and absolute log2 fold change >1) using the enrichR R package (version 3.1) together with the Reactome pathways database [23]. For GSEA/enrichment analysis, results with *p*-value/padj < 0.05 were considered significant.

### 2.4. Statistical Analysis of Flow Cytometry Data

For the statistical analysis of the flow cytometry data, non-parametric Kruskal–Wallis analysis and the two-stage linear step-up procedure of Benjamini, Krieger, and Yekutieli post-test or the Mann–Whitney U test were used as indicated. *p*-value/padj < 0.05 was considered significant.

## 3. Results

### 3.1. Invasiveness of Bladder Cancer Is Associated with Higher Immune Infiltration but Poorly Explains Tumor Immune Microenvironment Heterogeneity

**Figure A1:**
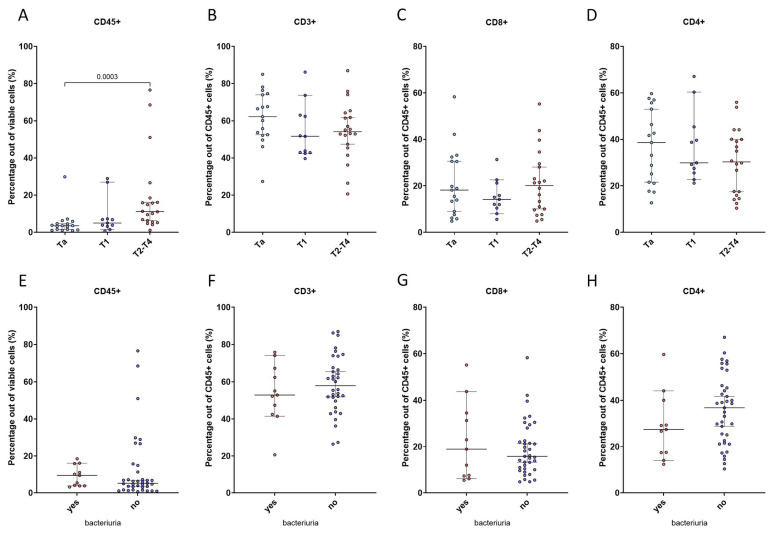
Infiltration of CD45^+^ immune cells out of all viable cells and frequency of T cell populations out of CD45^+^ immune cells in tumor samples stratified according tumor stage (**A**–**D**) and bacteriuria (**E**–**H**).

**Figure A2:**
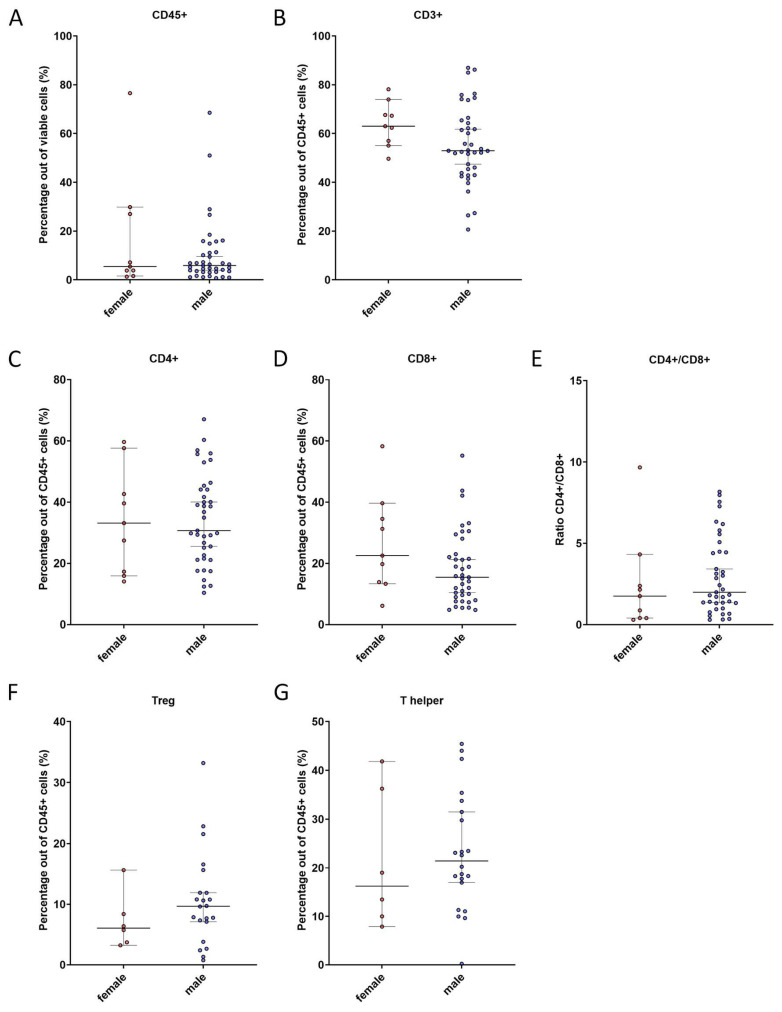
Infiltration of CD45^+^ immune cells out of all viable cells and frequency of T cell populations out of CD45^+^ immune cells in tumor samples according to sex (**A**–**D**,**F**,**G**). Ratio of CD4^+^/CD8^+^ T cells (**E**) Statistical significance was determined by Kruskal–Wallis test followed by two-stage linear step-up procedure of Benjamini, Krieger, and Yekutieli post-test.

We investigated the overall immune infiltration, as well as the proportion of T cells, CD8^+^ T cells, Th, and Tregs out of CD45^+^ cells in tumor biopsies from patients with bladder cancer stratified according to NMIBC/MIBC, as well as in control bladder tissue from patients without bladder cancer (gating strategy in Figure 1A). As expected, immune infiltration was significantly higher in both NMIBC and MIBC compared to control tissue (Figure 1B). In line with previous findings, muscle-invasive tumors were associated with higher immune infiltration (Figure 1B) [15,24]. However, no difference in the immune cell composition was detected between NMIBC and MIBC, as the proportion of CD3^+^, CD8^+^, CD4^+^, Th, or Tregs out of all CD45^+^ cells was not significantly different (Figure 1C–G). Stratifying according to tumor stage yielded similar results (Figure A1). At the same time, a large variation was seen among the NMIBC and MIBC samples, respectively. Furthermore, we did not observe any differences in immune composition in relation to bacteriuria status prior to surgery or in relation to sex (Figure A1 and Figure A2). Taken together, invasiveness was associated with overall immune infiltration in bladder cancer, but the NMIBC and MIBC dichotomy could not explain the heterogeneity within the tumor immune compartment.

### 3.2. Molecular Subtypes of Bladder Cancer Harbor Distinct Immune Microenvironments

**Figure 2:**
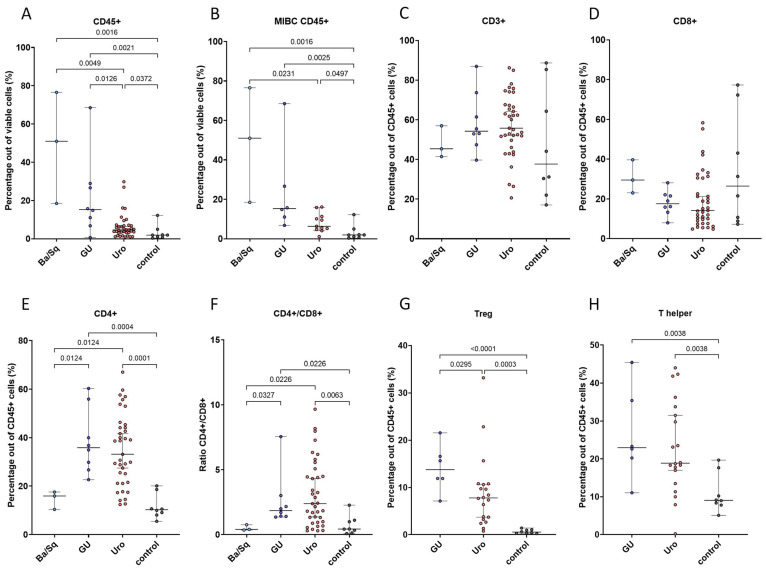
Frequency of immune cells and T cell populations in tumor samples classified according to the Lund Taxonomy molecular subtypes. Infiltration of CD45^+^ immune cells among all viable cells (**A**). Immune infiltration in MIBC samples only (**B**). Percentages of T cells, CD4^+^ T cells, and CD8^+^ T cells out of all CD45^+^ cells (**C**–**E**). Ratio of CD4^+^/CD8^+^ T cells (**F**). Percentages of Tregs and Th cells out of CD45^+^ cells (**G**,**H**). Statistical significance was determined through the Kruskal–Wallis test followed by the two-stage linear step-up procedure of Benjamini, Krieger, and Yekutieli post-test.

**Figure A3:**
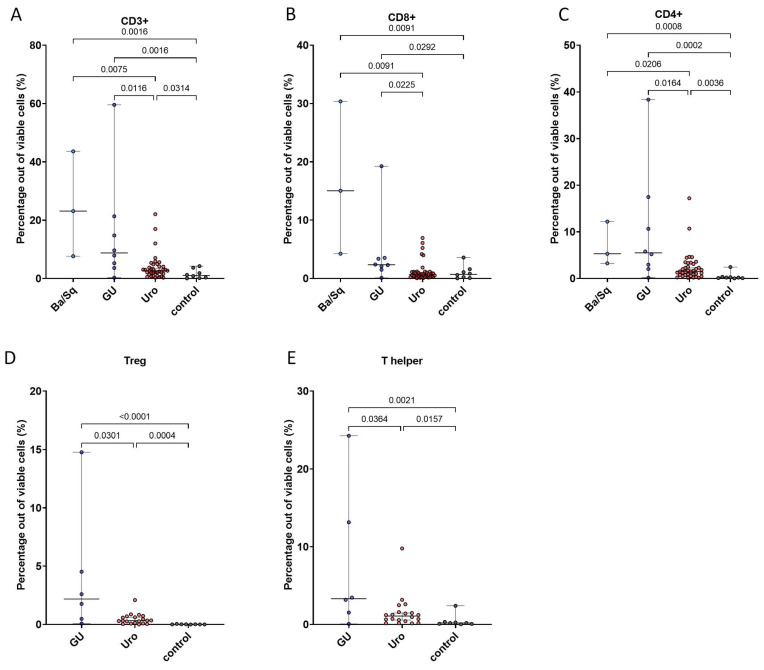
T cell populations in tumor samples classified according to the Lund Taxonomy molecular subtypes. Percentage of T cells (**A**), CD8^+^ T cells (**B**), CD4^+^ T cells (**C**), Tregs (**D**), and Th (**E**) out of all viable cells. Statistical significance was determined by Kruskal–Wallis test followed by two-stage linear step-up procedure of Benjamini, Krieger, and Yekutieli post-test.

Response to immunotherapy varies between molecular subtypes, and to better understand the immune environment underpinning these differences, we stratified tumor samples according to the Lund Taxonomy, which depicts the cancer cell phenotype independent of infiltration, and we analyzed the immune infiltration and abundance of T cell subsets. Immune infiltration was significantly higher in the Ba/Sq and GU subtypes compared to the Uro subtype (Figure 2A), and the same trend was seen among MIBC samples only (padj = 0.0608 GU vs. Uro) (Figure 2B). T cell infiltration (CD3^+^ cells out of viable cells) was also higher in the Ba/Sq and GU subtypes compared to the Uro subtype (Figure A3). In contrast, no statistically significant difference in the proportions of T cells or CD8^+^ T cells within the immune compartment were detected between the molecular subtypes (Figure 2C,D). Interestingly, the proportion of CD4^+^ T cells within the immune compartment, as well as the CD4/CD8 ratio, were higher in the GU and Uro subtypes compared to the Ba/Sq subtype (Figure 2E,F). Moreover, upon further analysis of the CD4^+^ T cells, it was shown that Tregs, but not Th cells, were significantly enriched within the immune compartment of the GU subtype compared to the Uro subtype (Figure 2G,H). Similarly, the proportion of Tregs out of all cells was higher in the GU subtype compared to the Uro subtype (Figure A3). Taken together, although the immune infiltration and the proportions of immune cells varied within each molecular subtype, statistically significant differences were detected.

### 3.3. Exhaustion Gene Profile Enriched in CD8^+^ T Cells from GU Tumors

**Figure 3:**
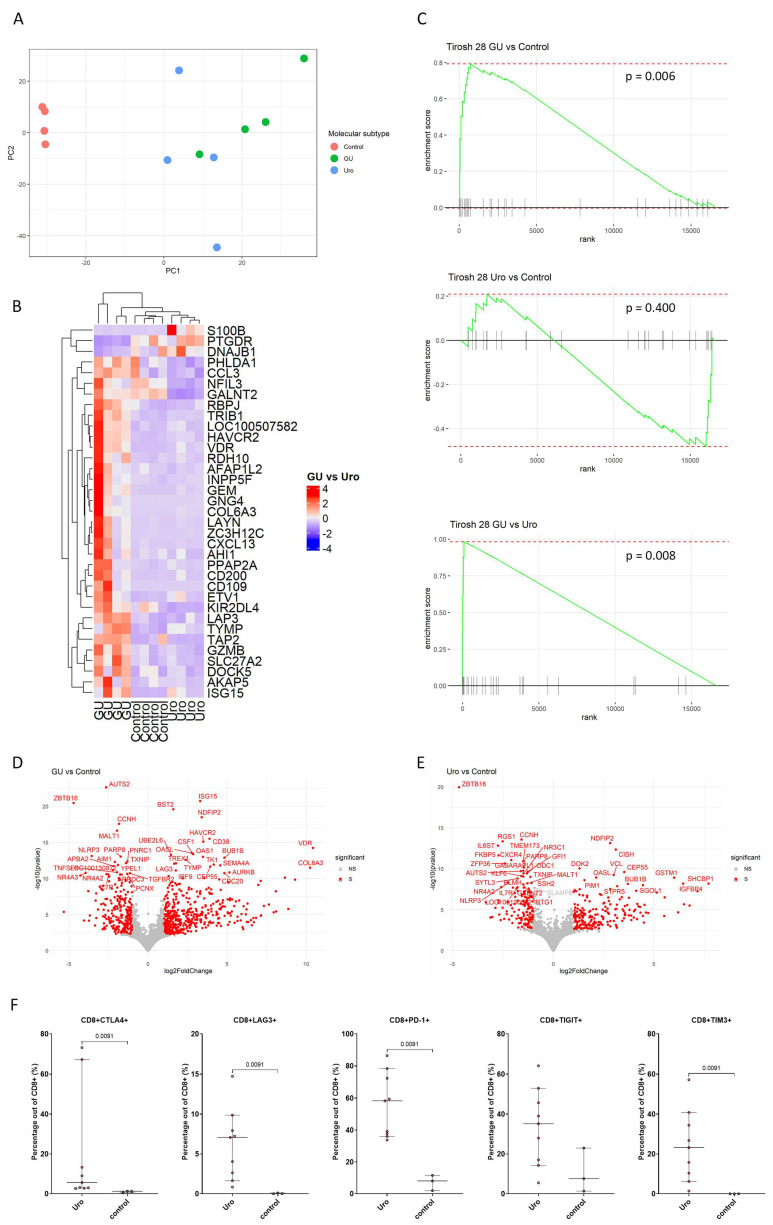
Transcriptional analysis of CD8^+^ T cells from non-malignant bladder tissue and from GU and Uro tumor biopsies. Principal component analysis (**A**). Heatmap of differentially expressed genes between CD8^+^ T cells from GU and Uro subtypes (**B**). GSEA using an exhaustion profile (Tirosh et al.’s 28-gene exhaustion profile) [25] (**C**). Volcano plot with the differentially expressed genes (GU vs. control) marked in red, with the top 40 annotated (**D**). Volcano plot with the differentially expressed genes (Uro vs. control) marked in red, with the top 40 annotated (**E**). Percentage of CD8^+^ cells expressing the exhaustion markers CTLA-4, LAG3, PD-1, TIGIT, or TIM-3 measured by flow cytometry (**F**). Statistical significance assessed by Mann–Whitney U test.

**Figure A4:**
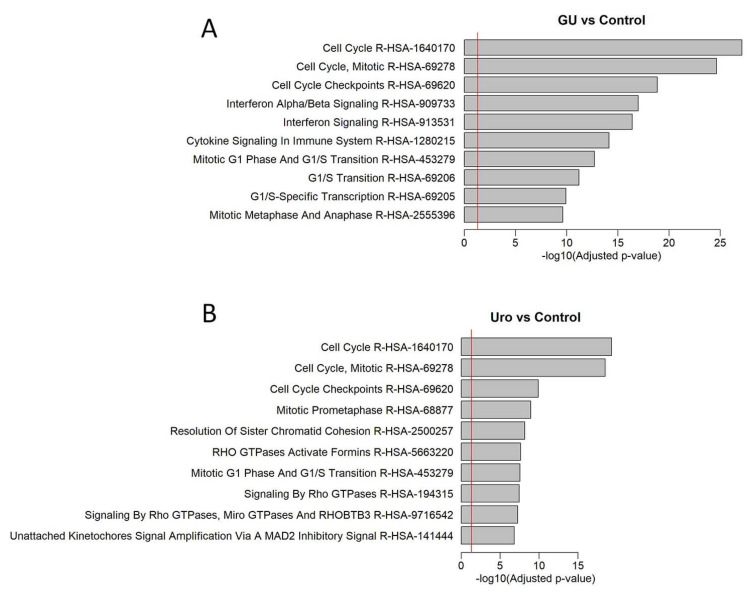
Pathway analysis of differentially expressed genes between CD8^+^ T cells from GU subtype and the control (**A**) and Uro subtype and the controls (**B**). padj = 0.05 marked in red.

To further investigate CD8^+^ T cells from the different molecular subtypes, we sorted and RNA sequenced CD8^+^ T cells from bladder tumor biopsies and control bladder tissues. After filtering out low-quality samples, adequate sample numbers were obtained for CD8^+^ T cells from control tissue (*n* = 4), GU (*n* = 4) and Uro tumors (*n* = 4). Principal component analysis revealed that the CD8^+^ T cell populations grouped according to control/cancer and, to some degree, according to molecular subtype (Figure 3A). Differential gene expression analysis resulted in 35 genes expressed at significantly different levels between GU and Uro tumors, several of which were related to T cell exhaustion, such as *LAYN*, *CXCL13*, and *HAVCR2* (Figure 3B). To further investigate T cell exhaustion, GSEA using an exhaustion profile was performed [25]. The results revealed that CD8^+^ T cells from GU tumors were significantly enriched for the exhaustion profile when compared to CD8^+^ T cells from Uro tumors and control tissue, whereas no difference was observed between CD8^+^ T cells between Uro tumors and control tissue (Figure 3C). Furthermore, several genes were found to be differentially expressed by CD8^+^ T cells from GU and Uro tumors compared to the control tissue, and pathway analysis revealed that these genes were mostly related to cell cycle and immune signaling (Figure 3D,E and Figure A4). Interestingly, CD8^+^ T cells in GU tumors showed higher expression of the gene *VDR,* encoding the vitamin D receptor, previously associated with reduced exhaustion [26], compared to both Uro tumors and controls (Figure 3D). For the Uro subtype, we further investigated the presence of exhaustion markers on the cell surface. Contrary to the findings on the transcriptomic level, we observed a significant increase in CTLA-4, LAG-3, PD-1, and TIM-3-positive CD8^+^ T cells in the Uro tumors compared to the controls. Sample numbers for the other subtypes were not adequate to perform statistical analysis, as the assessment was performed on fresh samples at a timepoint when the subtype was unknown (Figure 3F).

## 4. Discussion

In this study, we investigated the immune microenvironment in bladder tumors using flow cytometry and RNA sequencing. We show that the level of immune infiltration differs between NMIBC and MIBC as well as between molecular subtypes. Importantly, the immune microenvironment composition was significantly different between the molecular subtypes, with lower CD4^+^ T cell frequencies in Ba/Sq and GU tumors having a higher proportion of Tregs than Uro tumors. These differences could impact the functional response to cancer therapies, especially immunotherapy, and they should be considered when designing future clinical trials.

As bladder cancer has traditionally been divided according to the invasiveness of the disease, and because this stratification largely decides whether organ-sparing or radical treatment is suitable, we initially stratified the samples accordingly. We found that immune infiltration varied greatly between patients and that muscle-invasive disease was associated with higher overall immune infiltration, as demonstrated previously using DNA methylation analysis and tissue microarrays (TMAs) [15,24,27]. However, the observed variation in the proportion of T cell subsets (out of CD45^+^ cells) could not be explained by the MIBC/NMIBC dichotomy. This suggests that while the amount of immune infiltration increased with increasing tumor stage, there are changes in the composition of the immune compartment that relate to underlying biology rather than invasion depth. Furthermore, incidence rate and survival in bladder cancer have been shown to differ between the sexes [28]. For example, increased mortality has been observed for women within the first two years after diagnosis [29,30]. Moreover, sex differences have been implicated in anti-tumor immune responses in lung cancer [31]. In our study, we did not observe any significant differences in immune infiltration or composition in relation to sex, thus suggesting that the reported differences in bladder cancer may be related to infiltrating myeloid cells [32] or have other or additional underlying causes. It should be noted, though, that the incidence rate for bladder cancer is higher among males; hence, there are more samples from males in the study.

Bladder cancer molecular subtyping is a stratification system with higher resolution, and we show that the median immune infiltration was highest in the Ba/Sq subtype, followed by the luminal subtypes GU and Uro, which is in line with previous findings [15,33,34]. While MIBC comprises tumors of the Uro, Ba/Sq, and GU subtypes, the Uro subtype is by far the most common subtype among NMIBCs. By restricting the analysis to only MIBC cases, we demonstrate that the increased immune infiltration in Ba/Sq and GU tumors compared to Uro tumors still persists, indicating that the difference in NMIBC/MIBC prevalence does not explain the observed difference. We also observed an increase in infiltration of T cells in Ba/Sq and GU tumors compared to Uro tumors and this could potentially also be true for other immune populations. The agreement with previous findings on immune infiltration based on larger patient cohorts and using bulk RNA seq [7] or tissue staining [15] supports that our sample collection accurately represents the different molecular subtypes.

By further disentangling the immune compartments, we showed that the proportion of CD4^+^ T cells out of all CD45^+^ cells is higher in GU and Uro tumors compared to Ba/Sq tumors and controls. A comparison of the CD4^+^/CD8^+^ T cell ratio between the subtypes showed differences between subtypes, with Ba/Sq having the lowest ratio. We furthermore showed that Tregs are more abundant (proportion out of all cells) in GU compared to Uro tumors, which is in line with previous findings based on staining of tissue microarrays [15]. Importantly, our approach allowed us to also investigate the relative abundance of Tregs within the immune compartment. We demonstrated that the proportion of Tregs out of CD45^+^ cells is higher in GU tumors compared to Uro tumors. Although it has previously been suggested that Tregs in bladder cancer, unlike many other solid cancers [35], can be associated with better prognosis [11], the immunosuppressive role of Tregs is well-established, and the correlation could be due to Treg infiltration being associated with the infiltration of cells that contribute to the anti-tumor immune response. Despite the association, the consensus is that Tregs should be inhibited/eliminated in order to promote anti-tumor immune responses. This is, in fact, a strategy currently being pursued in clinical trials [36]. Targeting Tregs might constitute an additional therapeutic axis to further improve the anti-tumor immune response in bladder cancer, and, given the relatively high Treg abundance, GU tumors could be particularly responsive.

For the CD8^+^ T cells, we observed no significant difference in frequency in CD45^+^ cells among the different subtypes, but CD8^+^ T cells from GU, Uro, and control non-malignant bladder tissue (from patients not suffering from bladder cancer) were further compared using sequencing. Enrichment analysis revealed an upregulation of cell-cycle-related pathways in CD8^+^ T cells from both GU and Uro tumors compared to the control, suggesting local proliferation of T cells as a potential source of tumor-infiltrating T cells. Interestingly, CD8^+^ T cells in GU tumors also showed a higher expression of the vitamin-D-receptor-encoding gene, and triggering this receptor with the active form of vitamin D was recently shown to inhibit the transcription of several exhaustion markers in cytotoxic T cells [26]. In the study, treatment of patients with non-small-cell lung cancer with Rocaltrol (active vitamin D_3_) decreased the expression of PD-1, TIM-3, and TIGIT and resulted in cytokine production associated with anti-tumor immunity. In addition to *VDR*, several exhaustion-related genes were upregulated in GU tumors compared to both Uro tumors and control tissues, whereas no significant difference was observed between Uro tumors and control tissues. In contrast, the exhaustion markers analyzed at the protein level using flow cytometry were indeed shown to be higher in Uro tumors compared to the control, suggesting that some degree of exhaustion of CD8^+^ T cells also occurs in the Uro subtype. This observed discrepancy in protein- versus transcriptomic-level expression could perhaps be explained by the large variation in expression of the investigated exhaustion markers within the Uro cohort and that the limited number of samples sequenced expressed low levels of these. Only one GU tumor was available for flow cytometry analysis, and no comparisons could thus be made of the protein expression of exhaustion markers. In contrast to our findings, Oh et al. observed no significant difference in the frequency of exhausted CD8^+^ T cells (their CD8_ENTPD1_ cluster formed upon analysis of single-cell RNA seq data) in bladder tumors compared to control bladder tissues [37]. However, their control was bladder tissue adjacent to the tumor, which, unlike the control tissue used in the present study, could be affected by the tumor, and this might explain why we observed a significant increase in CD8^+^ T cell exhaustion in bladder tumors compared to control non-malignant tissue. Taken together, our results suggest that CD8^+^ T cell exhaustion can be a phenomenon in both Uro and GU subtypes.

Given the differences in immune infiltration and composition between subtypes, different immunomodulatory interventions might be warranted for specific subtypes. Of note, there are now ongoing efforts to investigate the utility of stratifying patients according to molecular subtypes clinically (GUSTO trial: NIHR128103). More work in this area could reveal subtype-specific immune modulatory strategies, which could improve patient stratification and treatment selection.

The current study is not without limitations. No systematic exclusion of patient samples was made based on patient comorbidities, and any effects thereof cannot be ruled out. Also, it should be noted that the sample cohort comprised a higher proportion of MIBC compared to the incidence rate in the population. The reason for this is that the size of some tumors, predominantly NMIBC, was inadequate to allow for a detailed analysis of T cell subsets. Furthermore, the limited number of samples from subtypes other than the Uro subtype decreased the statistical precision in the comparisons between subtypes. Similarly, the number of control bladder tissue samples was limited, as these are obtained during the rare event of performing bladder surgery for reasons other than bladder cancer. This control tissue was selected to ensure that the control samples were truly not affected by bladder cancer, thus representing controls more similar to healthy bladder tissue compared to, e.g., tissue adjacent to the tumor. Thus, although a larger cohort is needed to validate our findings, the data related to the rare control material are considered valuable, and the results regarding the rarer molecular subtypes offer new and potentially useful information for these patient groups.

## 5. Conclusions

Molecular subtypes in bladder cancer differ in relation to immune infiltration and composition, suggesting that distinct biology and, thus, putative targets exist in the respective subtypes. In particular, targeting Tregs could provide an additional therapeutic avenue in the GU subtype, further boosting the effectiveness of immunotherapy. These results support the further implementation of molecular subtypes in clinical trials for established as well as explorative therapies to tailor treatments and achieve improved survival.

## Figures and Tables

**Table 1:** Cohort characteristics. Variables depicted as absolute numbers and percentage of all tumor samples. Spread described as interquartile range (IQR). Samples were collected between the years 2018 and 2022.

**Patient Samples**	***n***	**%**
Tumor	48	86
Control	8	14
**Healthy controls**		
Age, median (IQR)	55 (46–59.5)	
Sex	***n***	**%**
Male	**4**	50
Female	**4**	50
**Tumor samples**		
Age, median (IQR)	76 (68.5–79.5)	
Sex	***n***	**%**
Male	39	81
Female	9	19
**Stage (WHO 1999)**	***n***	**%**
Ta	17	35
T1	11	23
T2	8	17
T3	9	19
T4	3	6
**Grade**	***n***	**%**
G1	1	2
G2	15	31
G3	32	67
**Clinical nodal stage**	***n***	**%**
N0	41	85
N1	3	6
N2	2	4
N3	2	4
**Metastasis**	***n***	**%**
MX	17	35
M0	29	60
M1	2	4
**Molecular subtype**	***n***	**%**
Basal/Squamous	3	6
GU	8	17
Uro	37	77

## Data Availability

The raw data supporting the conclusions of this article will be made available by the authors on request.

## Appendix A

**Table A1:** Antibodies used for flow cytometry analysis and sorting of T cell populations from tumor and control bladder tissues.

Staining Agent	Clone	Supplier
BD Horizon™ Fixable Viability Stain 620	-	BD biosciences
FITC Mouse Anti-human CD45	HI30	BD biosciences
PerCP-Cy5.5 Mouse Anti-human CD19	HIB19	Biolegend (San Diego, CA, USA)
PerCP-Cy5.5 Mouse Anti-human CD20	2H7	BD biosciences
PerCP-Cy5.5 Mouse Anti-Human CD56	B159	BD biosciences
PerCP-Cy5.5 Mouse Anti-Human CD66b	G10F5	BD biosciences
PerCP-Cy5.5 Mouse Anti-Human CD14	MφP9	BD biosciences
PerCP-Cy5.5 Mouse Anti-Human CD16	3G8	BD biosciences
BV785 Mouse Anti-Human CD3	SK7	Biolegend
PE-Cy7 Mouse Anti-Human CD8	RPA-T8	BD biosciences
BV605 Mouse Anti-Human CD4	RPA-T4	BD biosciences
BV421 Mouse Anti-Human CD127	HIL-7R-M21	BD biosciences
APC Mouse Anti-Human CD25	M-A251	BD biosciences
PE Mouse Anti-Human CTLA4	L3D10	Biolegend
PerCp.Cy 5.5 Mouse Anti-Human TIGIT	A15153G	Biolegend
PE-Cy7 Mouse Anti-Human PD-1	EH12.2H7	Biolegend
BV510 Mouse Anti-Human CD4	RPA-T4	Biolegend
BV711 Mouse Anti-Human TIM3	7D3	BD biosciences
BV785 Mouse Anti-Human CD3	SK7	Biolegend
R718/APC-R700 Mouse Anti-Human LAG-3	T47-530	BD biosciences
APC-Cy7 Mouse Anti-Human CD8	SK1	BD biosciences
AF700 Mouse Anti-Human CD4	RPA-T4	BD biosciences
BV785 Mouse Anti-Human CD8	RPA-T8	Biolegend
BV510 Mouse Anti-Human CD3	SK7	Biolegend
